# Human Settlement and Landscape Anthropization of Remote Oceanic Islands: A Comparison between Rapa Nui (Pacific Ocean) and the Azores (Atlantic Ocean)

**DOI:** 10.3390/plants12112089

**Published:** 2023-05-24

**Authors:** Valentí Rull

**Affiliations:** 1 Botanic Institute of Barcelona, Spanish National Research Council (CSIC), Pg. Migdia s/n, 08038 Barcelona, Spain; vrull@csic.es or valenti.rull@icp.cat; 2 Institut Català de Paleontologia Miquel Crusafont, Universitat Autònoma de Barcelona, Bld. ICTA-ICP, C. Columnes s/n, Cerdanyola del Vallès, 08193 Barcelona, Spain

**Keywords:** Rapa Nui, Easter Island, Azores Islands, human settlement, anthropization, flora, vegetation, landscape, paleoecology, palynology

## Abstract

The flora and vegetation of oceanic islands have been deeply affected by human settlement and further landscape modifications during prehistoric and historical times. The study of these transformations is of interest not only for understanding how current island biotas and ecological communities have been shaped but also for informing biodiversity and ecosystem conservation. This paper compares two oceanic insular entities of disparate geographical, environmental, biological, historical and cultural characteristics—Rapa Nui (Pacific Ocean) and the Azores Islands (Atlantic Ocean)—in terms of human settlement and further landscape anthropization. The similarities and differences between these islands/archipelagos are discussed considering their permanent colonization, the possibility of earlier settlements, the removal of the original forests and the further landscape transformations leading to either full floristic/vegetational degradation (Rapa Nui) or major replacement (Azores). This comparison uses evidence from varied disciplines, notably paleoecology, archaeology, anthropology and history, to obtain a holistic view of the development of the respective socioecological systems from a human ecodynamic perspective. The most relevant issues still to be resolved are identified and some prospects for future research are suggested. The cases of Rapa Nui and Azores Islands may help set a conceptual basis for ocean-wide global comparisons among oceanic islands/archipelagos.

## 1. Introduction

Human settlement and landscape anthropization of oceanic islands is a topical issue that is able to provide small-scale models for understanding the effects of anthropogenic pressure on ecological dynamics, thus informing biodiversity and ecosystem conservation [1,2,3]. Anthropogenic extinctions accelerated five centuries ago by the development of new navigation technologies, which made overseas travel significantly safer and more efficient. This allowed European explorers to travel and colonize almost any corner of the planet, which caused irreversible ecological modifications involving deep landscape changes and the extinction of native species [4]. According to the IUCN (International Union for Conservation of Nature) Red List of Threatened Species (www.iucnredlist.org, accessed on 22 May 2023), which is the most reliable source to assess the magnitude and patterns of recent anthropogenic extinctions, approximately 900 species have gone extinct worldwide during the last 500 years as a consequence of human activities, such as overhunting, deforestation, habitat destruction, increased land use, replacement by introduced species or the introduction of alien pathogens. Most of these extinctions occurred in the Americas (30%), Africa (20%) and the Pacific islands (30%), followed by Eurasia, Australia and the Indo-Malayan region (<10% each) [4]. These numbers may be even larger if we consider extinctions predating European contact, mainly on oceanic islands [5], which are especially sensitive to human pressure due to size and connectivity constraints. In addition to direct effects of human actions, islands are very sensitive to the introduction of alien invasive species, which may constitute a major and persistent threat for autochthonous biodiversity.

Not only extinctions but also profound ecosystem and landscape disruptions have been caused by the prehistoric and historical discovery and colonization of oceanic islands. Terrestrial insular ecosystems have been particularly affected by vegetation disturbance and the introduction of plants and vertebrates from elsewhere [6,7,8,9,10,11,12,13]. Human impacts on insular vegetation vary depending on the particular features of the target islands and archipelagos, and the cultural particularities of the colonizer people. In general, original plant communities have been changed to support human life at variable intensities, ranging from full ecosystem degradation to the total or partial replacement of autochthonous vegetation with species of varied geographical and ecological origins, which can be considered true (intentional or nonintentional) experiments of ecosystem engineering [14]. The less frequent situation has been the persistence of preanthropic vegetation due to little or no human impact.

**Figure 1:**
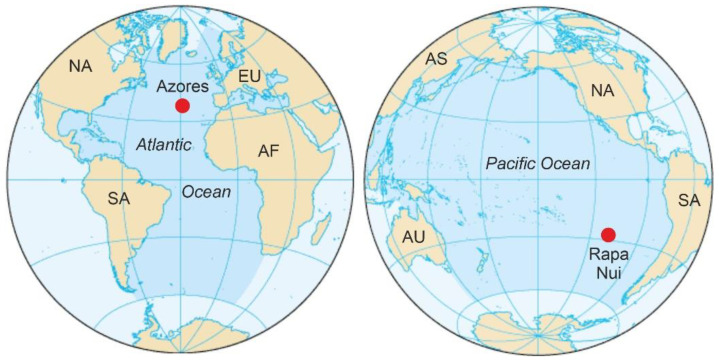
Locations of Rapa Nui and the Azores Islands (red dots) in the Pacific and Atlantic oceans, respectively. AF, Africa; AS, Asia; AU, Australia; EU, Europe; NA, North America; SA, South America.

This review compares two cases that are now actively discussed in the scientific literature and represent contrasting scenarios from geographical, environmental, biological, historical and cultural perspectives. One case is the subtropical Rapa Nui island (also known as Easter Island) lying in the southeastern Pacific (Figure 1), which was permanently settled by ancient Polynesians, arriving from the west between 800 CE and 1200 CE. The other case is the North Atlantic temperate Azores archipelago, which was permanently settled by the Portuguese, navigating westward, in the mid-15th century. Both Rapa Nui and the Azores were heavily anthropized after settlement but in very different ways due to the different timing and the cultural contrast between the respective settlers. Emphasis is placed on floristic turnover and vegetation change as major elements of landscape dynamics. The similarities and differences among the spatiotemporal patterns of discovery, colonization and landscape transformation of Rapa Nui and the Azores are discussed and compared. The main aim is to provide a conceptual framework that could be useful for comparing human settlement and anthropization patterns across ocean-wide and global scales.

The review begins with a brief account of the main current features of the target islands (Section 2), followed by thematic sections subdivided into discovery and settlement (Section 3), deforestation and landscape transformation (Section 4) and the potential influence of climatic changes (Section 5). Finally, a comparison is made between Rapa Nui and the Azores on the basis of the information provided in these sections (Section 6), followed by some general conclusions (Section 7) and the most relevant prospects identified for future research (Section 8).

## 2. Rapa Nui and the Azores

The highly isolated and heavily anthropized Rapa Nui island and Azores archipelago have the same geological origin but differ in many environmental and biological features. Both island complexes were originated from hotspot volcanism, the first in the Pleistocene (0.8 million years ago or Ma) [15] and the second between the Late Miocene (8.2 Ma) and the Pleistocene (0.3 Ma), although the exact dates for each individual island are still debated [16,17,18]. This section concentrates on the main geographical, climatic, floristic and vegetational features that differentiate Rapa Nui from the Azores. The prehistoric and historical ecological variations will be discussed in the following sections within the context of human settlement, further spatiotemporal land-use patterns and environmental change. Emphasis will be placed on palynological reconstructions using lake and swamp sediments situated inside volcanic craters, which are described in this section, in combination with the available historical and archaeological information. Rapa Nui is an excellent example of full island degradation, while the Azores archipelago is a representative case of replacement, that is, the substitution of the original vegetation by newly assembled plant communities composed of species introduced from elsewhere [3].

### 2.1. Rapa Nui

**Figure 2:**
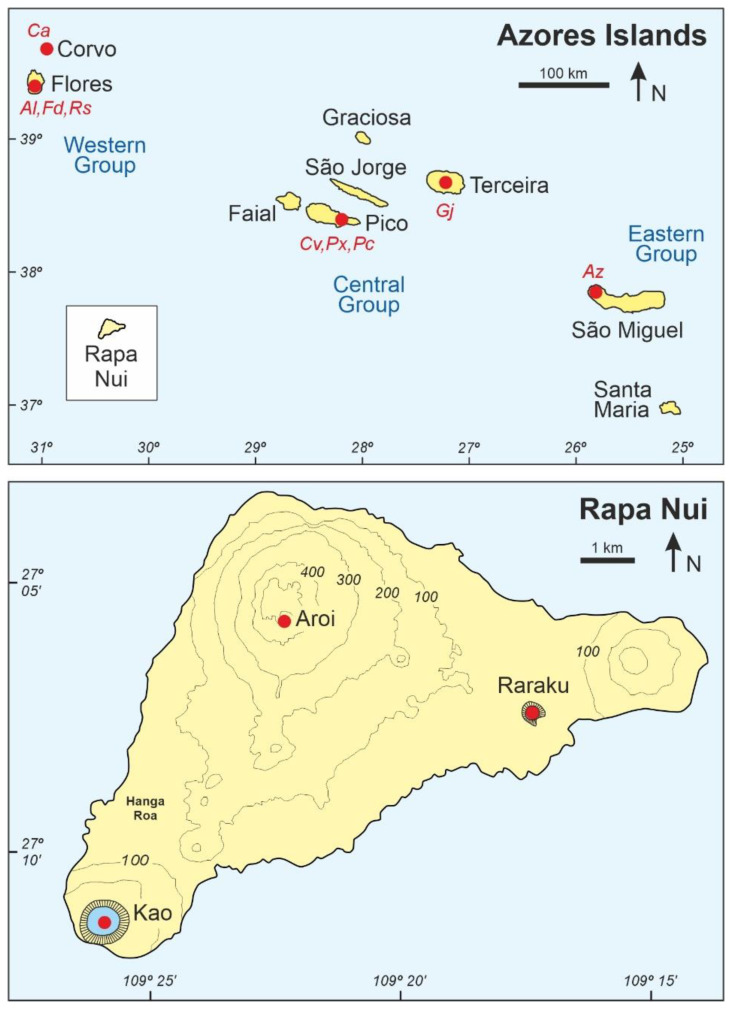
Sketch-map of the Azores archipelago (**above**) and Rapa Nui (**below**) at different scales to facilitate representation of the localities mentioned in the text. Rapa Nui (164 km^2^), of intermediate size between Flores (141 km^2^) and Faial (173 km^2^), is also represented in the upper panel (white box) at the same scale of the Azores islands for comparison. Red dots are the coring areas, some of which contain more than one coring site (Al, Alagoinha mire; Az, Lake Azul; Ca, Lake Caldeirão; Cv, Lake Caveiro; Fd, Lake Funda; Gj, Lake Ginjal; Pc, Pico bog; Px, Lake Peixinho; Rs, Lake Rasa). Details are available in Ref. [20] for Rapa Nui and Refs. [14,21,22,23] for the Azores.

Rapa Nui is a small (164 km^2^) subtropical southeastern Pacific island (27° 06′ 52′′ Lat S; 109° 25′ 31′′ Long W) (Figure 2) that has been considered among the most remote inhabited places on Earth. The island has a triangular shape and the maximum elevation is 511 m. The nearest continental coasts are from Chile (South America), situated ~3700 km east and—if the small (0.15 km^2^) islet Mottu Motiro Hiva (Salas y Gómez), situated 400 km E-NE is not considered—the nearest island is Pitcairn (Polynesia), which is located ~2100 km west. Geographically, Rapa Nui is part of eastern Polynesia, but politically it belongs to Chile. Approximately 45% of the Rapa Nui inhabitants consider themselves as Rapanui, that is, descendants of the Polynesian colonizers (the ancient Rapanui), whereas the others are of Chilean origin, although genetic mixing between these groups is common [19].

**Figure 3:**
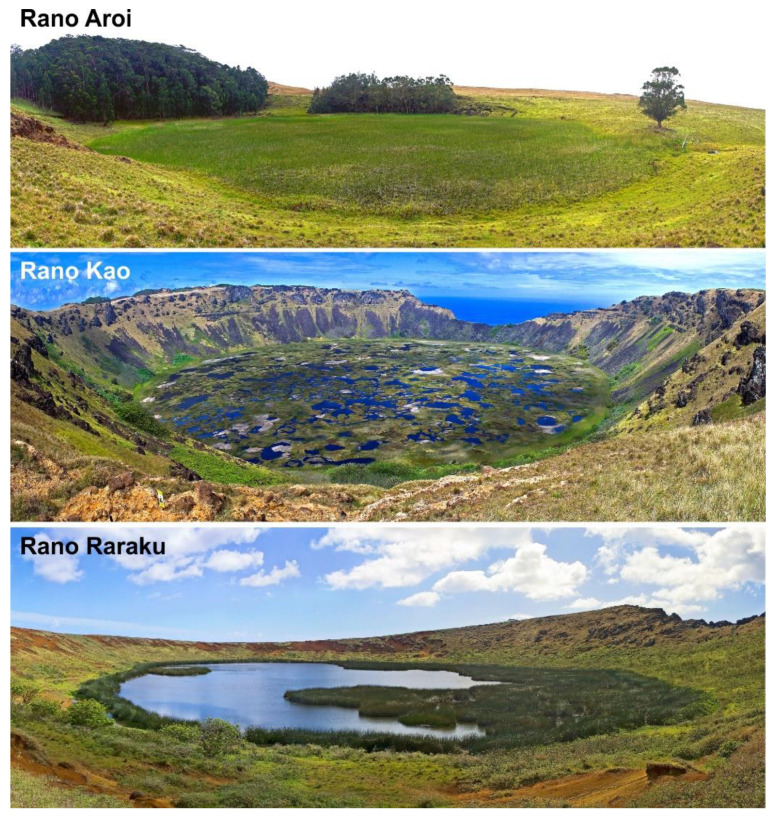
Panoramic views of the three craters containing aquatic sediments useful for paleoecological reconstruction in Rapa Nui. Rano is the indigenous name for a crater with a freshwater swamp or a lake. Note the absence of primeval palm forests and the dominance of grasslands. In Rano Aroi, the small tree stands are *Eucalyptus* (Myrtaceae) plantations introduced from Australia. In Rano Raraku, the darker aquatic vegetation is dominated by the autochthonous sedge *Scirpus californicus*. Photos: V. Rull.

The climate is subtropical, with small seasonal temperature variations due to the oceanic influence. The annual average temperature is 21 °C with a gentle seasonal range of 5–6 °C on average, between 18 °C in the Austral winter (July–September) and 23–24 °C in the Austral summer (January–March). The total annual rainfall ranges between 1100 and 1300 mm. The average seasonal variability ranges between minima of 70–80 mm/month (November–December) and maxima of 100–130 mm/month (April–June). Potential evapotranspiration is approximately 850–950 mm/year; therefore, the climatic hydrological balance (precipitation-evapotranspiration ratio) is above 1, which means that there is no water deficit during a typical year [24]. Some preliminary studies suggested a temperature decrease of −0.85 °C and 175 mm per 100 m elevation [25]. Precipitation seasonality is controlled by the interplay between the Sotuh Pacific Anticyclone (SPA), the South Pacific Convergence Zone (SPCZ) and westerly storms tracts [26]. The interannual variability is relatively high, especially for precipitation, and the influence of the El Niño Southern Oscillation (ENSO) on these trends is currently debated [27,28,29]. In Rapa Nui, the combination of a crater and a freshwater wetland is known as “rano”, and the most studied ranos from a palynological point of view are the Aroi fen and the lakes Kao and Raraku (Figure 2 and Figure 3).

According to the most recent update [30], the extant wild vascular flora of Rapa Nui is composed of 201 species (21 ferns, 1 gimnosperm and 179 angiosperms), of which only 50 (25%) are native and the remaining 151 (75%) are introduced. Half of the native species (21 ferns and 4 angiosperms) are considered to be endemic. Regarding vegetation types, no traces of native palm forests remain on the island, which is mostly covered by grass meadows (74%), with a few patches of planted forests (6%) and shrublands (2%) and a few cultivated areas (4%). The remaining 14% of the surface corresponds to urban areas and uncovered/barren lands [31].

### 2.2. The Azores

The North Atlantic temperate archipelago of the Azores Islands (37°–40° Lat N; 25°–31° Long W) is situated ~1400 km west of Portugal (Europe), with Madeira (~900 km SE) as the nearest island. The archipelago is composed of three groups of islands: the Western Group (Corvo and Flores), the Central Group (Faial, Graciosa, São Jorge and Terceira) and the Eastern Group (Santa Marta and São Miguel) (Figure 2). The total surface is 2351 km^2^ and the nine major islands mentioned range from 17 km^2^ (Corvo) to 745 km^2^ (São Miguel). The maximum elevations are between 587 m (Santa Maria) and 2350 m (Pico). The Azores are part of the biogeographical region known as Macaronesia, together with the subtropical Madeira and Canary Islands, and the tropical Cape Verde Islands, all of which are closer to the African coasts. Politically, the Azores are an autonomous region of Portugal and the society is typically Portuguese.

**Figure 4:**
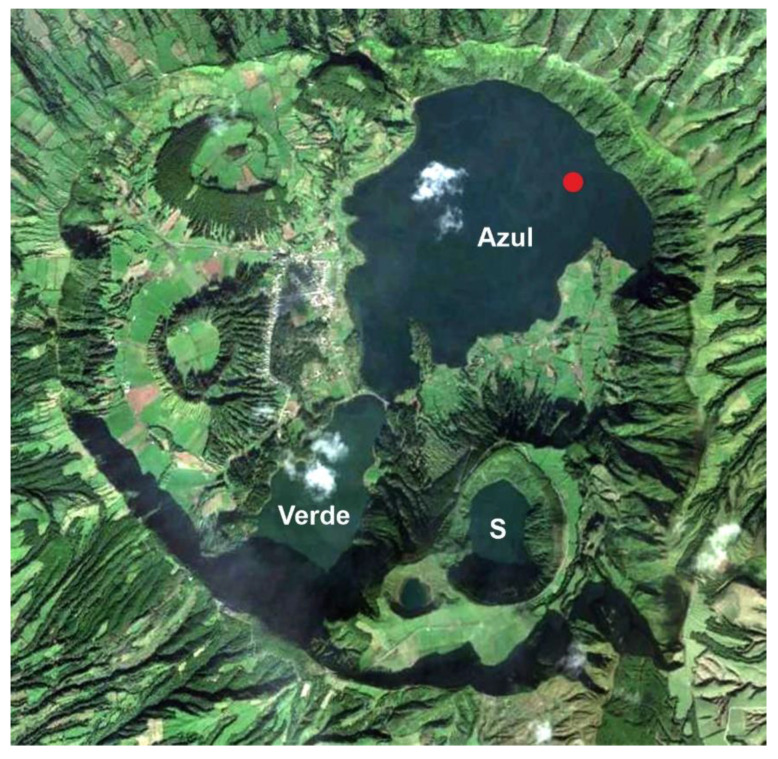
Google Earth image of the crater Sete Cidades, on Azorean São Miguel Island, and its three lakes (S, Santiago). The red dot in Lake Azul is the coring locality. The darker green patches are planted *Cryptomeria* (Cupressaceae) forests, introduced from Japan. Modified from Ref. [14].

The Azorean climate is temperate oceanic and is characterized by mild temperatures with small annual variations, a strong seasonal precipitation regime and significant interannual variability. At lower elevations, the average annual temperatures range from 16.7 °C (Terceira) to 17.5 °C (Corvo and Santa Maria) and the total annual precipitation is between 775 mm (Santa Maria) and 1716 mm (Flores). However, at higher elevations, average annual temperatures may decrease to 10.8 °C (900 m) and 6.2 °C (2000 m), and total annual precipitation may be over 4000 mm [32]. Maximum precipitation occurs between November and January and minimum values are recorded between June and August, a seasonality that is controlled by the displacement of the Azores anticylone [33,34]. The interannual temperature and precipitation regimes are influenced mainly by the North Atlantic Oscillation (NAO) and the Atlantic Multidecadal Oscillation (AMO) [34,35]. The palynological records of the Azores archipelago are from Corvo (Lake Caldeirão) and Flores (lakes Funda and Rasa, and Alagoinha mire) in the Western Group, Pico (lakes Caveiro and Peixinho, and Pico bog) and Terceira (Lake Ginjal), in the central Group, and São Miguel (Lake Azul) in the Eastern Group (Figure 2 and Figure 4).

Regarding floristic composition, 1110 species of vascular plants have been reported outside cultivation, of which 811 (73%) are considered part of the true Azorean flora, which includes all permanent and self-sustaining populations, whereas the remaining 299 (27%) are considered non established [36]. Among the true floristic elements, only 24% (197 species) are indigenous and 76% (562 species) are introduced (69% naturalized and 6% established). Among the 197 indigenous taxa, 66 (30%) are endemic. Regarding vegetation, the most extensive type are pastures (42% of the total surface), followed by forests (35%) and crops (14%); the remaining surface corresponds to urban (5%) and barren areas (4%) [37]. Native vegetation represents 13% of the total and only 2.5% corresponds to primeval forests [32].

## 3. Discovery and Settlement

In this paper, a differentiation is made among terms and concepts such as discovery, settlement and contact, along with their different types, as shown in Table 1. Historical records report the arrival of the first Portuguese colonizers to Azores (Santa Maria and São Miguel islands) in 1432 CE and consider 1449 CE as the official settlement date [38]. However, the archipelago had already been discovered, as some of its islands already appear in maps from 1339 CE under the names of Corvinaris (Corvo) and Caprara (São Miguel) [39]. No indisputable archaeological evidence exists of former settlements. The situation is different in Rapa Nui, where abundant and well-dated archaeological and anthropological evidence exists for permanent settlement of the Polynesian Rapanui culture since at least 1200-1300 CE [40,41]. In this case, the first historical records date from 1722, when the island was visited by Dutch explorers, which is known as the European contact, and was the beginning of the end of the prehistoric Rapanui culture [42,43]. The possibility of human settlement before the dates documented by historical and archaeological evidence has been suggested for both Azores and Rapa Nui. This possibility is called here the early settlement hypothesis.

### 3.1. Rapa Nui

The first paleoecological (palynological) studies on Rapa Nui sediments reported major island deforestation from ~800 CE, which was linked to the arrival of Polynesian colonizers [44,45]. Several dating problems related to the occurrence of stratigraphic gaps and sediment mixing cast doubts on the reliability of this chronology [46]. Mann et al. [47] suggested that Rapa Nui could have been settled, perhaps transiently or intermittently, by Polynesian hunter-gatherers several centuries before the 1200 CE Rapanui establishment, when forests were cleared, but these authors did not provide evidence for this assumption. Later, Mieth & Bork [48], using edaphic evidence of landscape modification, proposed an earlier phase of low-impact human occupation, beginning at 800 CE, before the 1200 CE deforestation. This low-impact early settlement phase, ranging between approximately 800 CE and 1200 CE, is present in many Rapa Nui chronologies using a variety of archaeological and paleoenvironmental evidence [20,49,50].

**Figure 5:**
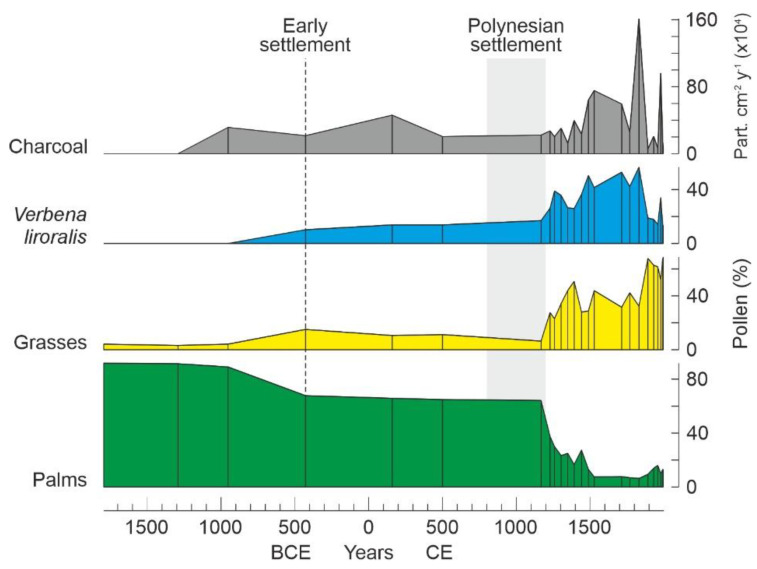
Simplified pollen diagram from Lake Raraku for roughly the last 2.5 millennia showing the early appearance of *Verbena* (450 BCE) coinciding with a moderate palm forest decline and grassland expansion. Raw data from Ref. [51].

The recent finding of paleoecological evidence compatible with human presence in sediments older than 800 CE has suggested that the first settlement could have occurred earlier. Especially significant is the finding of pollen from *Verbena litoralis* (Verbenaceae), a plant of tropical American origin that is presently widespread all over the island, in Lake Raraku sediments. This anthropochorous (dispersed by humans) plant, locally known as “puringa” and characteristic of agricultural and ruderal disturbed sites, was previously considered to have been introduced on the island in post-Columbian times (1492 CE), but is actually present in the Raraku sediments, in a continuous and abundant fashion, from 450 BCE to the present [51]. The appearance of *V. litoralis* pollen coincided with the first fires and a forest decline coeval with a grassland expansion (Figure 5), which was interpreted in terms of anthropogenic disturbance, probably by small human groups of unknown origin, as formerly suggested by Mann et al. [47]. Also significant is the presence of pollen from the Iridaceae *Sisyringium* (probably *S. michrantum*) between 410 CE and 630 CE in the sediments of the Aroi bog. This plant is also of American origin and is now naturalized elsewhere. Other potential indications of human disturbance are the forest declines documented by 50–100 CE in Lake Kao [52] and by 1730-910 CE in Lake Raraku [53]. However, the lack of specific anthropogenic markers prevents sound assessment.

**Figure 6:**
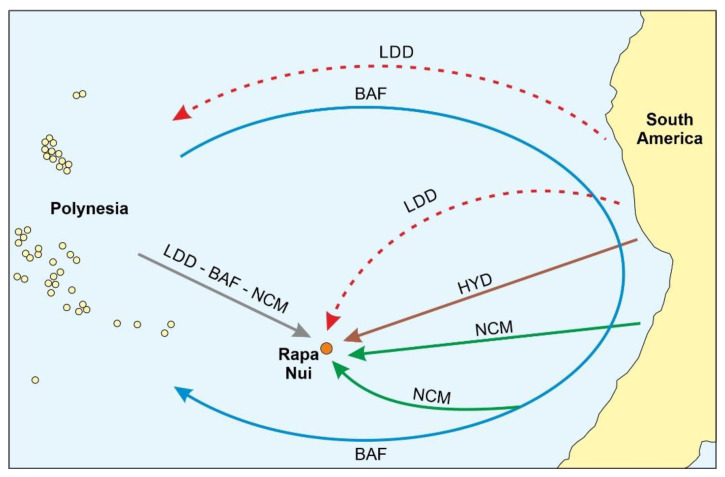
Four existing hypotheses for the arrival of the sweet potato (*Ipomoea batatas*) in Rapa Nui. According to the Long-Distance Dispersal hypothesis (LDD), sweet potato seeds could have arrived in Polynesian islands (or directly to Rapa Nui) by birds, winds or rafting, and then transported by humans to Rapa Nui. The back-and-forth (BAF) hypothesis implies the pre-Columbian arrival of Polynesians to South America, from where they could have transported the sweet potato to Polynesia and, from there, to Rapa Nui. The Heyerdahl (HYD) and the Newcomers (NCM) hypotheses propose the direct human transportation of sweet potato from South America. The difference is that the HYD hypothesis contends that the plants were carried by Amerindians before Polynesian settlement (400 CE), whereas the NCM hypothesis postulates that this occurred after Polynesian colonization and does not clarify whether the Amerindians arrived in Rapa Nui by themselves or were carried by Polynesians in their back-and-forth voyages. Modified from Ref. [20].

The sweet potato (*Ipomoea batatas*; Convolvulaceae), locally called “kumara”, is known to have been a fundamental food for ancient Polynesian societies, especially for Rapa Nui, where it has been suggested that the flourishment of the ancient Rapanui society would have not been possible without this plant [54]. The sweet potato was domesticated in tropical America >6000 years ago [55], and the oldest Polynesian records date to 1000 CE (almost 500 years before the Columbian arrival to America, in 1492 CE), as documented by carbonized fragments found in the Cook Islands [56]. On Rapa Nui, carbonized fragments or starch grains of kumara have been found on sediments and archaeological sites since 1300–1400 CE, approximately 3–4 centuries before European contact [53,57]. Four hypotheses have been suggested to explain this fact: long-distance seed dispersal, back-and-forth traveling to America, the American origin of the first Rapa Nui settlers, and the arrival of American people between Polynesian colonization and European contact (Figure 6). After analyzing the multidisciplinary evidence supporting each of these hypotheses, Rull [58] concluded that all options remain open, which has been supported by more recent investigations [59,60,61,62,63].

Interestingly, pollen and macrofossil evidence from cultivated plants support a potential early colonization of Rapa Nui pointing toward a pre-Columbian American origin, which strongly suggests pre-European contact between the island and America, as proposed for the Polynesian region in general [64]. This seems to revive the former Heyerdahl’s hypothesis [65] of an initial settlement by Amerindian (or Native American) peoples followed by a Polynesian invasion. Although this hypothesis was declared dead a couple of decades ago after a thorough analysis of the available multidisciplinary evidence [66], the latest molecular analyses of human genomes suggest the presence of Amerindian people on Rapa Nui prior to Polynesian settlement [67,68]. Unfortunately, no archaeological evidence is available for these early settlers, which could have been removed by natural or anthropogenic agents (evidence clearing [20]) or hidden by recent sea-level rise [69,70]. It is possible that early settlements were local and ephemeral/intermittent, and the archaeological evidence left was scattered and easy to remove. Thus, the only signs of early human settlement would remain fossilized in the unaltered sediments of lakes and bogs. Further research on molecular biomarkers would be of much help to resolve this conundrum.

### 3.2. Azores

**Figure 7:**
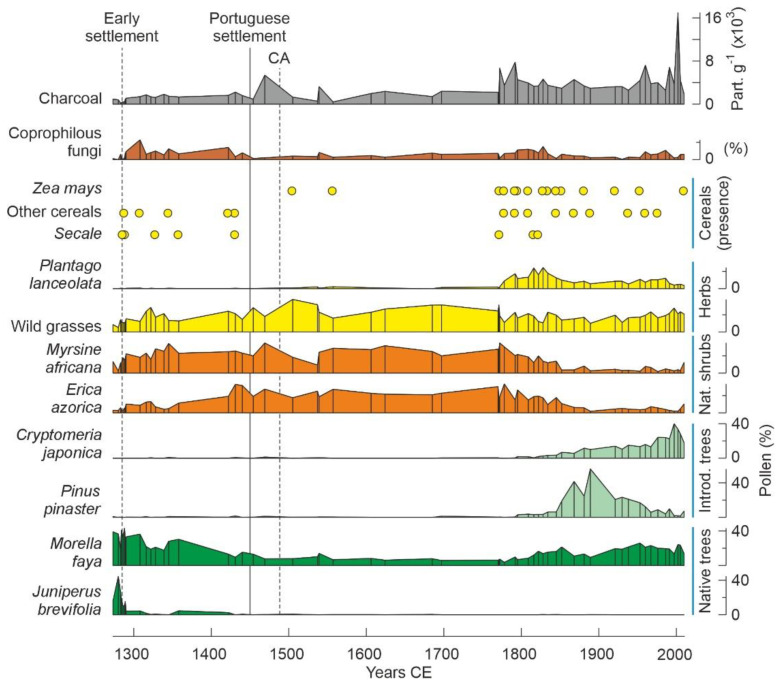
Summary palynological record from Lake Azul (São Miguel Island) showing the major elements in relation to early settlement and further anthropization trends. CA, Columbian arrival to America. Simplified from Ref. [14].

In the Azores Islands, any eventual settlement before Portuguese colonization in the mid-15th century may be considered an early settlement. As stated above, there is no archaeological evidence of this fact but paleoecological records are, once more, the most informative in this sense. The first palynological records documented significant vegetation changes, notably deforestation, just after Portuguese settlement, in the Corvo, Flores (Western group; WG) and Pico (Central Group; CG) islands [21,22]. But this will be discussed in detail in the next section, here the focus is on the possibility of earlier settlements. The first Azorean record of pre-Portuguese human impact was from São Miguel Island (Eastern group; EG) and consisted on palynological evidence for the decline of *Juniperus*, one of the main elements of the pristine forests, coeval with the first fires and the appearance of cereal pollen, notably from rye (*Secale*), along with spores from coprophilous fungi (notably *Sporormiella*) growing in the dung of herbivores [14] (Figure 7). These animals were absent from the archipelago, as the only mammals inhabiting the Azores before human settlement were bats [39]. This was considered to be sound evidence for local-scale deforestation around the coring site (Lake Azul), cereal cultivation and animal husbandry, and occurred by 1290 CE, approximately one century and a half prior to the official Portuguese settlement of the islands and half a century before their historical discovery, as documented in navigation charts. The geographical and cultural origins of potential early settlers remain unknown.

More recently, it has been proposed that the archipelago would have been settled much earlier, by the early Middle Ages (700–850 CE), at least 6–7.5 centuries before the Portuguese, and suggested that the first settlers would have been the Norse, with the aid of anomalous northeasterly winds and warmer temperatures [23]. These conclusions were based on new multiproxy analyses of lake sediments from five islands, two of the WG (Corvo, Flores), two of the CG (Pico, Terceira) and one from the EG (São Miguel), including the abovementioned Lake Azul record, which suggested the authors widespread and permanent transformations of terrestrial and aquatic ecosystems, including deforestation by logging and fire, and livestock grazing. As a consequence, the landscape would have already been deeply and extensively modified before Portuguese settlement [23]. This idea has been considered unlikely by other authors, who believed that the evidence is insufficient and the conclusions premature [71]. These critics emphasize that if the landscape would had been significantly anthropized in the Middle Ages, this should have been noted by the Portuguese colonizers, whose descriptions of natural systems, especially the flora and vegetation, were very detailed and accurate. In addition, a number of methodological and interpretation points were raised, including dating issues and alternative explanations for the occurrence of pollen from cultivated plants, charcoal and fecal lipids [71]. It is not the possibility of earlier human presence that is questioned but the fact that the Azores were already significantly and extensively anthropized prior to Portuguese arrival. These critiques were replied by the authors of the Norse hypothesis [72], although the new explanations provided did not satisfactorily address all points raised by the critics (Elias, pers. comm.) and left the debate open.

The Norse hypothesis has also been questioned by Rull [73], who emphasized the unavailability of detailed raw data and the inaccuracy of some representations, as well as the lack of sufficient early Medieval records. Indeed, the authors of the Norse hypothesis presented their results in a synthetic fashion with no access to detailed analyses. In the case of landscape anthropization, a major point of the debate, pollen records are critical evidence, but only summary diagrams are provided, which hinders detailed reconstructions of vegetation change and comparison with other similar records. Particularly significant is the lack of differentiation within forest categories, especially between native and introduced forests. In Lake Azul, this separation is possible because the detailed pollen diagram was published some years ago [14], but in others, this cannot be evaluated with the information provided. Another issue is the inaccuracy of some representations, including cereal pollen, one of the most important proxies for anthropogenic impact. Indeed, in the same Lake Azul record, *Secale* pollen is represented only in the 13th century in the form of scattered occurrences [23], but it truly occurred between the 13th and 19th centuries [14] (Figure 7). Additionally, other cereal pollen, including maize (*Zea*) and wheat (*Triticum*), were omitted [23], but were actually present in a continuous and consistent fashion from the 13th century to the present [14]. Due to the lack of raw data, it is not possible to know whether the same occurs in other records and proxies.

**Figure 8:**
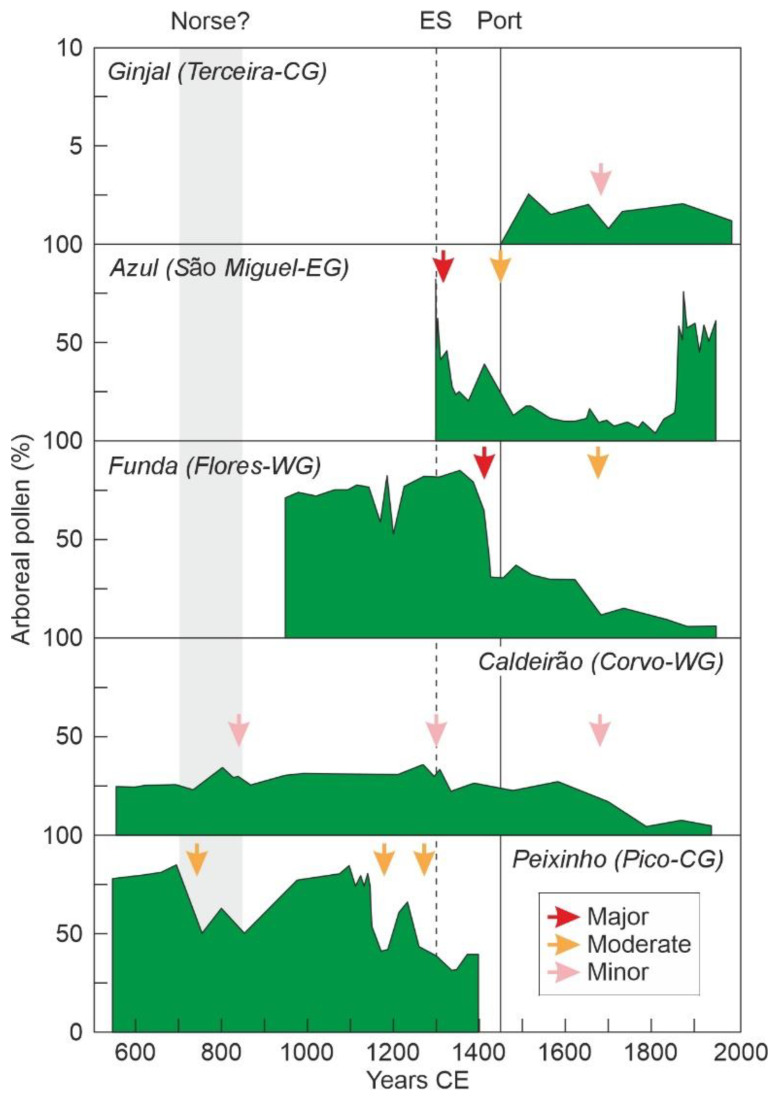
Arboreal pollen fluctuations in the five sequences analyzed by Raposeiro et al. [23], sorted chronologically. The arrows mark the main forest declines, according to the legend within the box. The gray band is the time interval proposed by the authors for the purported Norse settlement. Redrawn and simplified from the original. ES, early settlement; Port, Portuguese settlement.

Finally, of the five records utilized by the proponents of the Norse hypothesis [23], only two from Corvo (WG) and Pico (CG) extended back to 600 CE, whereas one (Flores; WG) began in 900–1000 CE and the other two, from São Miguel (EG) and Terceira (CG), started in 1300–1400 CE (Figure 8). Whatever the evidence for human settlement, these records are insufficient to demonstrate that the whole archipelago was settled before 900 CE, as only two records from central and western islands are available before that date. Additionally, the only significant forest decline recorded before 900 CE corresponds to one of these records (Pico; CG), but its attribution to human activities is doubtful, since fire would have been linked with volcanic activity, as documented by a tephra layer, and both coprophilous fungi and fecal lipids present in humans and other omnivores were absent until the 11th–12th centuries. Therefore, the only potential evidence of human impact before those dates remains debatable, and the only widespread evidence for human presence did not occur until the 13–14th centuries, as previously proposed by Rull et al. [14]. Publishing the detailed data for all proxies, improving representation and retrieving new records encompassing the whole Middle Ages is strongly suggested to evaluate the possibility of early settlements before those dates. What seems truly difficult to prove is that these potential earlier settlers significantly anthropized the landscape across the whole archipelago, as this should have been noticed by Portuguese settlers and is not recorded in the available palynological studies, which are extensive to the whole archipelago.

## 4. Deforestation and Landscape Transformation

In this case, several types of forest clearing will be considered according to their extent, intensity and mode. The term deforestation will be reserved for full forest removal, whereas partial forest clearing will be referred to as forest decline. Forest declines will be considered major if the pollen from forest trees decreases above 50% with respect to the initial abundance, moderate when the decrease is between 20% and 50% and minor if the decline is 20% or less. In addition, deforestation may be the result of a single forest clearing event (catastrophic deforestation) or the accumulation of minor to major forest declines (progressive deforestation), in which case, stepwise forest declines are called forest decline pulses or events. All the above forest clearing patterns may occur at local (single-site) or general (island-wide) ambits.

### 4.1. Rapa Nui

**Figure 9:**
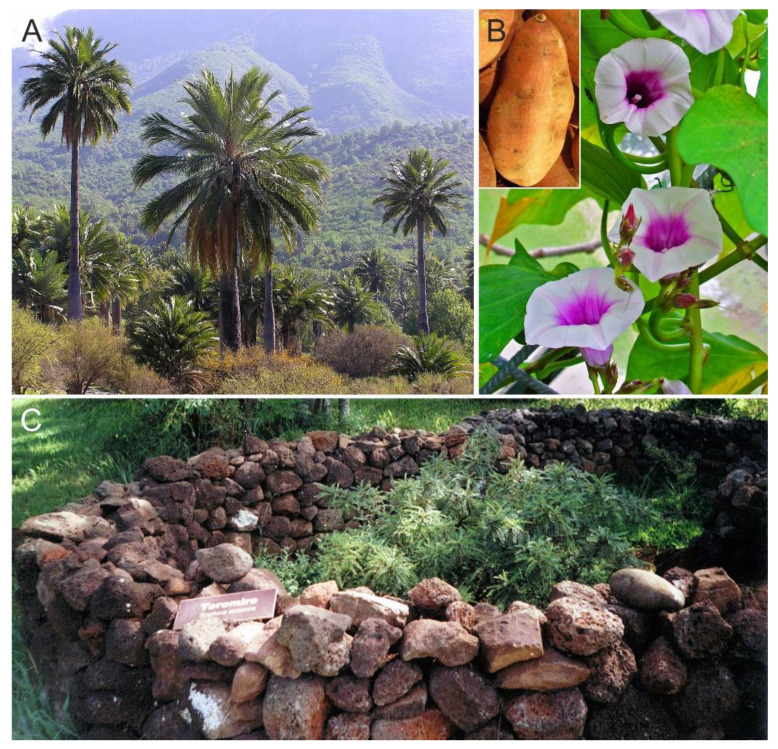
(**A**) The Chilean wine palm *Jubaea chilensis* growing in La Campana National Park, on the Pacific Chilean coasts (photo courtesy by A. Mieth). (**B**) Flowers and fruits of sweet potato (*Ipomoea batatas*) (https://commons.wikimedia.org/wiki/File:Ipomoea_batatas_002.jpg, accessed on 22 May 2023). (**C**) A modern reproduction of a “manavai” in the Botanical Garden of Hanga Roa, the capital of Rapa Nui (https://commons.wikimedia.org/wiki/File:ToromiroRapanui.jpg, accessed on 22 May 2023).

According to the available palynological studies—historical documents did not appear until European contact (1722 CE), when the island had already been deforested—the pristine Rapa Nui forests were largely dominated by an unknown, likely extinct, palm species that is only known by its fruits very similar to the Chilean wine palm (*Jubaea chilensis*) (Figure 9) and has tentatively named as *Paschalococcos disperta* [74,75]. Other minor forest components were *Sophora* (Fabaceae), *Triumfetta* (Tiliaceae), *Macaranga* and *Acalypha* (Euphorbiaceae), *Coprosma* (Rubiaceae), along with several unidentified Astraceae, Myrtaceae and Moraceae/Urticaceae [45]. These primeval dense palm forests (approximately 16–20 million palm trees in barely 160 km^2^, or 100–120 palms per km^2^) would have covered 70–80% of the island [76,77].

The classical literature on Rapa Nui usually considers a single type of forest removal, island-wide deforestation. However, the most recent studies have demonstrated that this process was heterogeneous in both space and time and that island-wide deforestation was actually the result of the accumulation of local minor to major forest declines and deforestation events [78]. As mentioned above, the first forest declines documented occurred long before the Polynesian settlement, but only two could be linked to the presence of humans because of the occurrence of pollen from anthropogenic plants of American origin. The first of these minor forest declines occurred in Raraku by 450 BCE [51], and the second took place in Aroi by 410–630 CE [79]. All other forest clearing events occurred during the development of the Rapanui culture, initiated with Polynesian settlement, and occurred in progressive (Kao, Raraku) or catastrophic fashion (Aroi).

**Figure 10:**
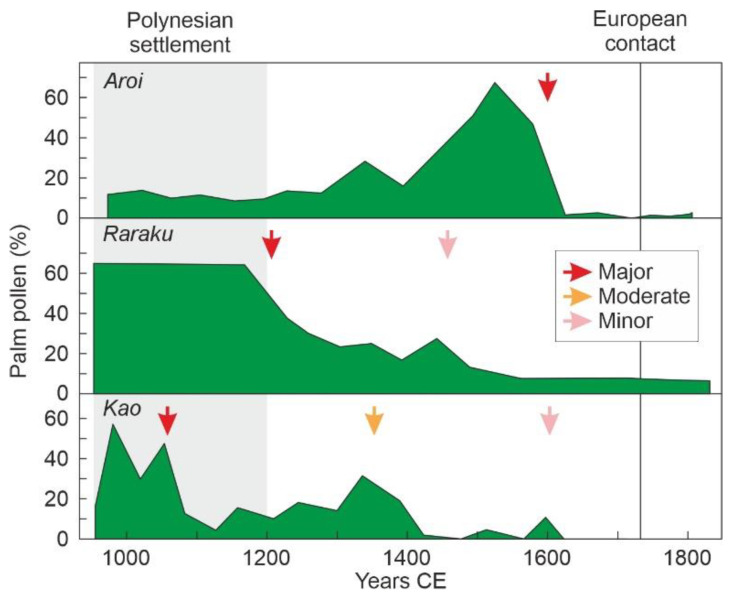
Trends of palm pollen in the three coherent and continuous sequences retrieved in Rapa Nui for the last millennium. The arrows mark thee main forest declines, according to the legend within the box. Redrawn and simplified from Ref. [78], using raw data from Refs. [51,80,81].

The first and last forest clearing events were recorded at Kao (Figure 10). The first of the events was a major local decline (1000–1050 CE), followed by a recovery and a moderate forest decline (1350 CE) that almost totally removed the Kao forests, a further modest recovery and a final minor but full deforestation event (1600 CE) [80]. The deforestation of Raraku took place in two steps, a major forest decline in 1200 CE and a minor but definitive forest decline at 1450 CE [51]. The only catastrophic deforestation was recorded at Aroi, where forests experienced a sustained increase between approximately 1300 CE and 1520 CE, when an abrupt deforestation event began that fully removed these forests. In all cases, except for the first Raraku event (1200 CE), forest declines coincided with charcoal peaks indicating that fire was a usual method for forest clearing. Spores of coprophilous fungi were only recorded in association with the last Kao event (1600 CE) and after, suggesting that significant grazing practices occurred only since those dates, which marked the full island-wide deforestation [78]. No coincidences were observed in the chronology or in the intensity of forest declines across the island, which highlights the significant spatiotemporal heterogeneity of Rapa Nui deforestation.

**Figure 11:**
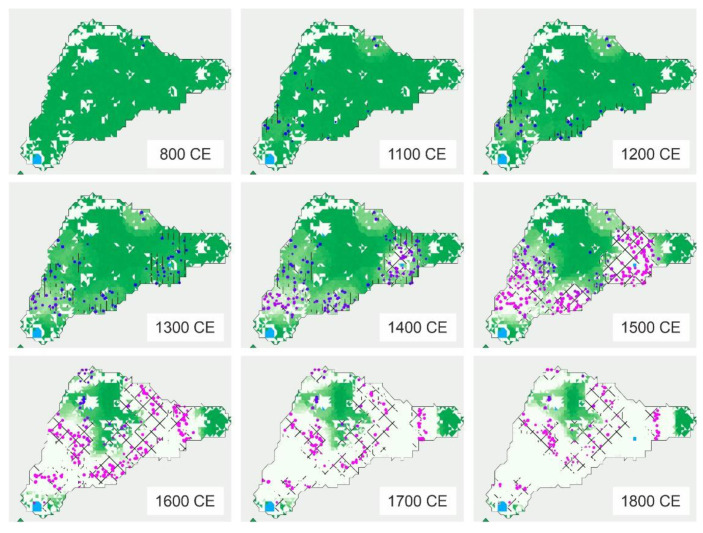
Forest (green areas) retreat and garden (blue and pink dots) proliferation in Rapa Nui during the last millennium, simulated with an agent-based model of human-resource interactions. The green intensity represents the density of palms, ranging from 0 (white) to 7 (dark green) thousand trees per cell. The color of dots is the resource preference, in percentage of trees vs. gardens, ranging from 20% (pink) to 80% (blue). Modified from Ref. [86].

Regarding cultivation, the first records correspond to the already mentioned micro- and macrofossil remains of sweet potato, dated to 1300–1400 CE [53,57]. Small-scale cultivation practices seem to have coexisted with forests since the beginning of Polynesian settlement [82]. However, as deforestation progressed, newly opened terrains were increasingly used for agriculture. The ancient Rapanui did not practice extensive cultivation or widespread irrigation, and crops were restricted to meter-scale gardens called “manavai” (Figure 9), whose water supply was heavily dependent on rainfall [25]. To minimize evaporation and erosion, the manavai were protected from wind and surficial runoff by rock walls, and the soils inside were covered by rocks to preserve heat, moisture and nutrients, a practice called lithic mulching [83,84]. Based on extensive field surveys, the first estimations yielded a surface of nearly 80 km^2^ of mulching area, approximately half of the island surface, during a period between about 1460 and 1860 CE [85]. The proliferation of gardening cultivation on newly deforested terrains during the development of the ancient Rapanui culture was modeled by Steiglechner & Merico [86] using multiproxy evidence (Figure 11). In addition to sweet potato, the main plants cultivated by the ancient Rapanui were “yam” (*Dioscorea alata*; Dioscoreaceae), “ipu kaha” or bottle gourd (*Lagenaria siceraria*; Cucurbitaceae), “taro” (*Colocasia esculenta*; Araceae), “ti” (*Cordyline fruticosa*; Asparagaceae), “mahute” or paper mulberry (*Broussonetia papirifera*; Moraceae) and “maika” or banana (*Musa* sp.; Musaceae), as documented by historical reports and pollen, phytoliths and starch grains from dryland soils and lake/swamp sediments [53,75,87,88,89,90].

Landscape anthropization did not stop with Rapanui deforestation and gardening but was exacerbated after European contact, as documented in historical records [42,43,54]. A major deterioration occurred in 1875 CE when the whole island was transformed into a ranch, mostly for sheep. If the former forest removal had eliminated the palm-dominated forests, intensive and extensive grazing removed most of the autochthonous plant species that remained. After a pause in which alien plant species were introduced, notably coconut trees (*Cocos nucifera*), a second and even more intense landscape degradation phase took place as a result of the reactivation of extensive livestock practices. In 1903 CE, the island experienced the worst vegetation deterioration of its entire history. The introduction of exotic species continued with the planting of *Eucalyptus* forest stands, which are still standing on the island (Figure 3), along with other species for reforestation and ornamental purposes, or to protect soils from erosion. A thorough account of the native and introduced species are provided in Refs. [30,75].

### 4.2. Azores

**Figure 12:**
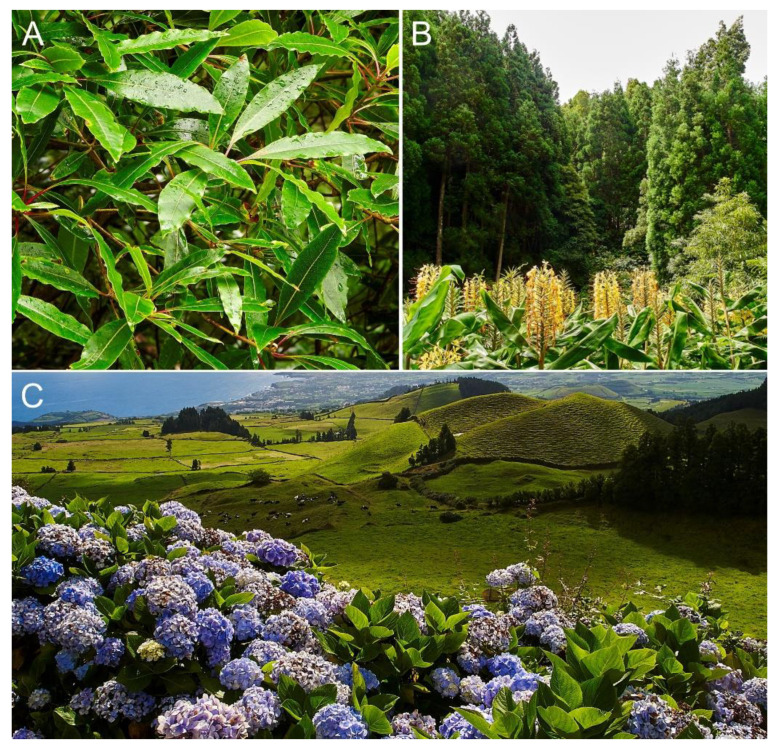
Native and introduced plants on São Miguel Island. (**A**) Leaves of *Laurus azorica* from a laurisilva remnant in Ruta do Tronqueiro. (**B**) Conifer forests of *Cryptomeria japonica* around Furnas, with a typical understory dominated by *Hedychium gardnerianum*. (**C**) Typical landscape from São Miguel Island, with extensive crops and pastures intermingled with *Cryptomeria* forests (dark green patches) and *Hydrangea macrophylla* in the foreground. Photos: V. Rull.

The historical accounts of Portuguese settlers and the available palynological records coincide in that the pre-settlement forests were of the laurisilva type, that is, temperate evergreen forests with a predominance of species with laurus-like coriaceous shiny leaves that favor mist condensation on their surface thus creating a humid microhabitat (Figure 12). These laurisilvas were dominated by *Laurus azorica* (Lauraceae), *Juniperus brevifolia* (Cupressaceae), *Prunus azorica* (Rosaceae) and *Morelle faya* (Myricaceae), with other trees and shrubs, such as *Frangula azorica* (Rhamnaceae), *Taxus baccata* (Taxaceae), *Picconia azorica* (Oleaceae), *Myrsine africana* (Myrsinaceae), *Viburnum tinus* (Adoxaceae), and the Ericaceae *Erica azorica*, *Vaccinium cylindraceum* and *Calluna vulgaris* [39,91]. Today, the vegetation is largely anthropogenic as a consequence of centuries of deforestation and the introduction of exotic species, leading to the replacement of most original forests, which are restricted to a few small sites that are under protection [92,93]. The landscape has been almost completely rebuilt in a process that has been subdivided into three main phases: a pre-settlement phase, an extractive phase and a transformative phase [21,91].

During the pre-settlement phase, between Portuguese landing (1432 CE) and the official settlement (1449 CE), several types of domestic animals (sheep, goats, pigs, horses) were released on the uninhabited islands in the hope that their populations increased naturally and facilitated the establishment of eventual future colonizers in the absence of wild herbivores. It has been documented that these introductions were highly successful but the impact on primeval forests was minimal and of local nature. The proliferation of the introduced livestock suggests that natural open vegetation types other than laurisilvas were available for these animals to graze. These open communities would have been upland grasslands [91] and are represented in the pollen diagrams by small percentages of Poaceae and other herbs and ferns [21]. During the extractive phase, initiated with Portuguese settlement, native forests were intensively exploited for construction (houses and ships), firewood and charcoal production, which is clearly reflected in paleoecological records by the decline of pollen from laurisilva elements, especially *Juniperus*, *Ilex* and *Morella* [14,21]. In these palynological records, *Laurus* pollen is poorly represented, but the local presence of this tree is documented by the occurrence of its characteristic stomata. Historical records document the overexploitation until exhaustion of many autochthonous plant species and communities—especially those useful for medical purposes, wood/charcoal extraction, fruit harvesting and the production of oils, dyes or materials for leather tanning, in which most of the abovementioned elements of native forests were involved—due to the colonialist mindset of the first settlers [91].

The unsustainable exploitation of natural resources and their ensuing exhaustion led to the introduction of new production systems imported from the continent, such as wheat, sweet potato or corn cultivation, along with the expansion of upland pastures for animal husbandry. This inaugurated the transformative phase, which was especially active during the last two centuries. Other important monocultures developed during this phase were sugar cane (*Saccharum officinarum*), grape (*Vitis vinifera*; Vitaceae), pepper (*Capsicum*; Solanaceae), pineapple (*Ananas comosus*; Bromeliaceae) and especially orange (*Citrus sinensis*; Rutaceae). A detailed account of the transformative phase on São Miguel Island is provided by Moreira [39] that could serve as a reference for the archipelago.

The first great agricultural success was the extensive cultivation of orange trees imported from China, whose maximum development was attained at the beginning of the 18th century and largely transformed the landscape and the economy of the island. Most forests were cut down to obtain the timber needed to make the boxes in which the oranges were exported. At that time, the Australian tree *Pittosporum undulatum* (Pittosporaceae) was introduced as a hedgerow species to protect orange crops from wind. The orange industry declined by 1830 CE for economic reasons and because of the action of fungal and insect parasites. By 1860 CE, large-scale pineapple cultivation was introduced to replace orange crops. Again, large amounts of timber were needed for pineapple exportation, but the island was largely deforested and had to be reforested using several *Pinus* (Pinaceae) species imported from Europe, *Cryptomeria japonica* (Cupressaceae) from Japan, and *Eucalyptus* spp. (Myrtaceae) and *Acacia melanoxylon* (Fabaceae) from Australia. The decline of the remaining native forest elements such as *Erica azorica* and *Myrsine africana*, along with the introduction of *Pinus pinaster* and *Cryotomeria japonica* by 1750–850 CE, are manifest in the lake Azul pollen record. These introduced tree species, together with *P. undulatum*, still dominate the forests of São Miguel (Figure 4), which occupy 25% of the island’s surface, whereas the other 75% is dedicated to human activities [91].

During the 18th and 19th centuries, many exotic ornamental species were also introduced to decorate public and private parks and gardens. Some of these species have become naturalized and are successful invaders, such as *Hedychium gardnerianum* (Zingiberaceae), which is native to the Himalayas and is dominant in the understory of many planted forests, mainly those of *Cryptomeria*. Another naturalized species that is widespread across the island, especially in ruderal habitats, is *Hydrangea macrophylla* (Hydrangeaceae), which is native to China and Japan (Figure 12). Among the trees, *P. undulatum* and *Clethra arborea* (introduced from Madeira) are the most successful invaders. Human pressure has been variable on the different islands, with Corvo and Flores being the least affected and São Miguel being among the most disturbed ones [37]. São Miguel Island has been taken here as an example of anthropogenic vegetation replacement [3]. The historical landscape transformation of São Miguel Island was described in detail by Moreira [39], and its paleoecological manifestation has been documented in the palynological study of Lake Azul [14].

In summary, the Azores Islands have not been fully deforested, and the extant forest formations are a mixture of native laurisilvas and newly assembled forest associations composed of alien tree species imported from elsewhere (Europe, Japan, Astralia), not occurring naturally in their places of origin. The main deforestation events documented palynologically occurred by 1290 CE (São Miguel) and shortly after Portuguese settlement (Pico, Flores) and involved the main native laurisilva elements [14,21]. In the other islands, although a number of minor to major forest declines have been documented (Figure 8), the lack of detailed pollen diagrams hinders more accurate interpretations.

## 5. Potential Influence of Climatic Changes

The idea that environmental changes heavily influence human societies until the point that they may trigger cultural demises is known as environmental determinism and is opposed by those who consider that cultural developments are a consequence of exclusively human actions (human determinism). The debate is maintained by the difficulty of demonstrating that a given cultural change is actually a consequence of a particular environmental shift. In most cases, these inferences are based on chronological coincidences, but unequivocal mechanistic demonstrations remain elusive [94]. This is the case for Rapa Nui and the Azores, where these chronological coincidences have been used as preliminary evidence for possible causal relationships. In both cases, the issue has been analyzed considering two main contexts, namely, the role of climatic/oceanographic conditions on the settlement of islands and the influence of environmental shifts on sociecological changes in already colonized islands.

### 5.1. Rapa Nui

The Polynesians arrived in Rapa Nui some time during the Medieval Climate Anomaly (MCA; 800–1300 CE), when warm/dry climates, higher sea levels and changes in wind patterns would have favored long-distance voyaging across the Pacific [95]. According to some paleoclimatic reconstructions, Polyensian settlement would have been facilitated by the occurrence of some climate windows for off-wind sailing between 800–820 CE and 1180–1250 CE, characterized by persistent westerly wind anomalies [96]. According to the same models, some windows also existed for off-wind sailing routes from South America and back in some periods between 910–930 CE and 1220–1260 CE. These figures are consistent with different hypotheses for Rapa Nui colonization but the authors favor Polynesian settlement between 1140 CE and 1250 CE, because its coincidence with current archaeological evidence [40,41]. This option is also supported by a recent biomarker study, accompanied by paleoclimatic reconstructions, pointing toward Polynesian settlement between 1150 CE and 1300 CE [97].

Other paleoclimatic/paleoceanographic models suggest that, in addition to winds and currents, climatic variability would have been a major driver in Pacific colonization ventures and propose precipitation as a key parameter in this regard [98]. The main drivers involved in Rapa Nui precipitation patterns are currently debated, with emphasis on the potential role of ENSO (El Niño Southern Oscillation) and the Pacific subtropical anticyclone [28,29]. A sustained drought documented on Rapa Nui between 500 CE and 1170 CE [51] partially overlapped the MCA and the 800–1200 CE settlement phase, which would be consistent with the occurrence of a significant climatic shift during the Polynesian settlement of Rapa Nui. Sear et al. [97] proposed that a similar drought between 800 CE and 1100 CE would have instigated initial eastward exploration from central Polynesia, culminating in Rapa Nui discovery and settlement roughly a century later. In addition to climate, regional volcanism and paleotsunamis that occurred during the last millennium have been suggested as potential modulators of transoceanic navigation, but this has not been studied in depth, and firm evidence is still unavailable [99].

In the classical literature, environmental shifts as drivers of ecological and cultural change on Rapa Nui have been systematically ignored by most archaeologists and explicitly dismissed by some paleoecologists. For example, Bahn & Flenley [100] argued that if palm forests survived the large temperature and moisture fluctuations experienced during the last 34,000 years, including the Last Glacial Maximum (LGM; occurred ~20,000 years ago), it would be unreasonable that they could have been annihilated by the comparatively lower climatic variability of the last millennia. Therefore, they concluded that the full island deforestation and the ensuing cultural collapse was a consequence of the overexploitation of natural resources by the ancient Rapanui society (possibly in combination with fruit-eating Polynesian rats), who caused their own demise (ecocide). On the other side of the debate, some proposed that intense and prolonged droughts that occurred during the Little Ice Age cold reversal (LIA; 1300–1850 CE) could have been instrumental for forest clearing [78]. Recent paleoecological research has identified one of these droughts (1520–1710 CE) coinciding with full island deforestation [51,101]. This suggested that forest removal could have been due to the combined action (amplification feedbacks) of humans and climate and further regeneration, which had been documented after previous clearing events, could have been hindered by the continued drought, along with the expansion of cultivation gardens [102].

**Figure 13:**
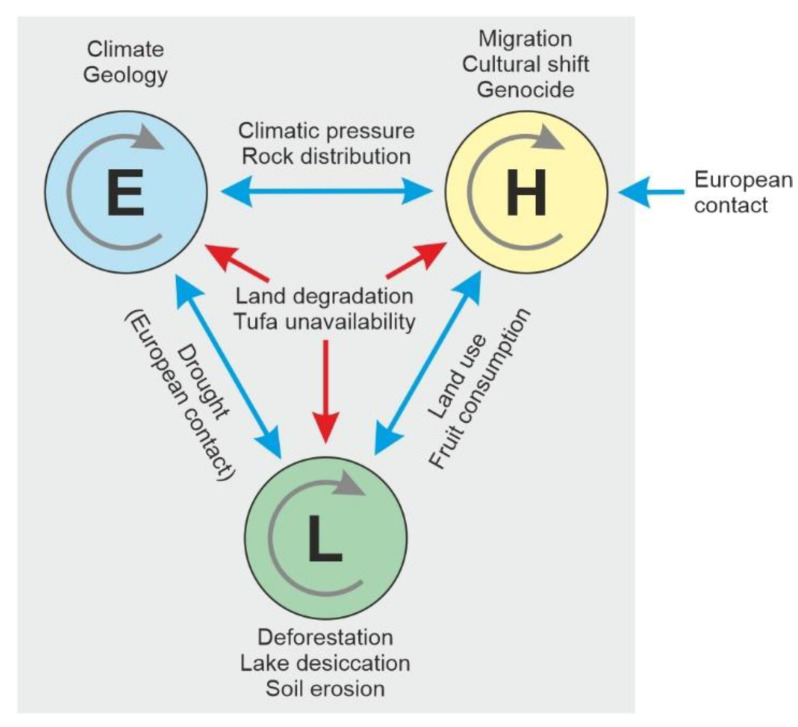
The EHLFS (environment-human-landscape feedbacks and synergies) framework as applied to Rapa Nui. E, environment; H, humans; L, landscape. After Ref. [103].

**Figure 14:**
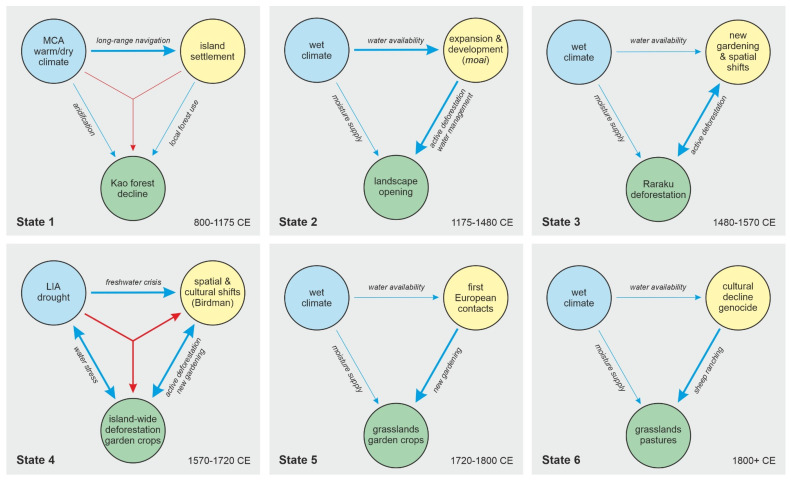
The different states of the EHLFS socioecosystem identified for the prehistory of Rapa Nui. Modified from Ref. [104].

This type of feedbacks and synergies between climate and humans had not been previously considered in the Rapa Nui literature and opened a new opportunity for a holistic approach in the analysis of socioecological systems, known as EHLFS (environment-human-landscape feedbacks and synergies) [103]. The EHLFS approach was used to define six states in the development of the Rapa Nui socioecosystem during the last millennium, under a human ecodynamic perspective [104]. These stages were characterized by spatiotemporal changes in the interactions between environmental (E), human (H) and landscape (L) subsystems (Figure 13 and Figure 14).

### 5.2. Azores

In their proposal for an early settlement by Norse people and the associated widespread landscape anthropization, Raposeiro et al. [23] utilized paleoclimatic simulations to infer the origin and the most likely routes of Norse colonizers. These authors concluded that, in early Medieval times, the temperature was anomalously high and the predominant winds reaching the Azores came from the NE, which would have facilitated the navigation from northern Europe, where the Norse lived, to the Azores. The problem is that these authors utilized paleoclimatic reconstructions from 850 CE onward, which do not match the period that they considered as the time of Norse settlement (700–850 CE), and therefore, their inferences are unwarranted. The same methodology was used to infer the predominant climatic conditions during the Portuguese arrival, which would have been fostered by a shift in wind patterns favoring the navigation between southern Europe (including Portugal) and the Azores [23].

**Figure 15:**
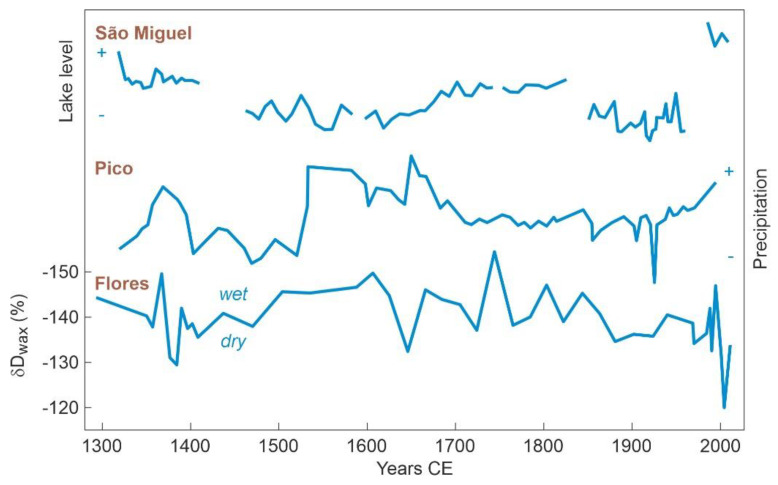
Hydroclimatic fluctuations as reconstructed in three lake sediment records from São Miguel [106], Pico [105] and Flores [107] islands using different proxies. Redrawn and composed from the original sources.

The first paleoecological reconstructions from the Pico and Flores islands [21] emphasized the lack of a clear relationship between the reconstructed forest trends and the available paleoclimatic records before and after Portuguese settlement [105] and attributed this fact to the highly maritime nature of the Azorean climate. Similar results were obtained in the São Miguel record [14], which suggested that humans would have been the main drivers of vegetation and landscape change. More recent paleoclimatic reconstructions identified the AMO and the NAO as the main mechanisms affecting the Azorean climate and discussed some potential relationships among climate changes, cultural and ecological shifts [106,107]. However, the available paleoclimatic records show significant departures, especially in relation to hydrological balance (Figure 15), and a consistent paleoclimatic framework for the whole archipelago is still to be attained. Additionally, an integrated socioecological assessment similar to the EHLFS approach has not yet been attempted. Therefore, in the present state of knowledge, the relationships between environmental, anthropogenic and landscape changes remain unknown. Significant additional efforts are still needed to clarify the potential role of past climate changes on socioecological systems.

## 6. Comparisons between Rapa Nui and the Azores

### 6.1. Present Conditions

Although Rapa Nui is more distant than the Azores from both the mainland and the nearest island, the species/area relationship, a key parameter in island biogeography since the time of McArthur & Wilson [108], is >3.5 times higher (Table 2). This feature was already recognized by Carine & Schaeffer [109], who emphasized the low number and extent of radiations and the scarcity of single-island endemics in the Azorean flora. These authors attributed these anomalous numbers, compared to the remaining Macaronesian and other Atlantic archipelagos, to the long-term stability of the Azorean climate, which would have inhibited speciation, and/or the incomplete knowledge of the Azorean flora. Later, the same authors reaffirmed the second explanation by recognizing many undiscovered genetically distinct taxonomic entities that drastically increased the diversity patterns and made the Azores more similar to other Atlantic archipelagos [92].

**Figure 16:**
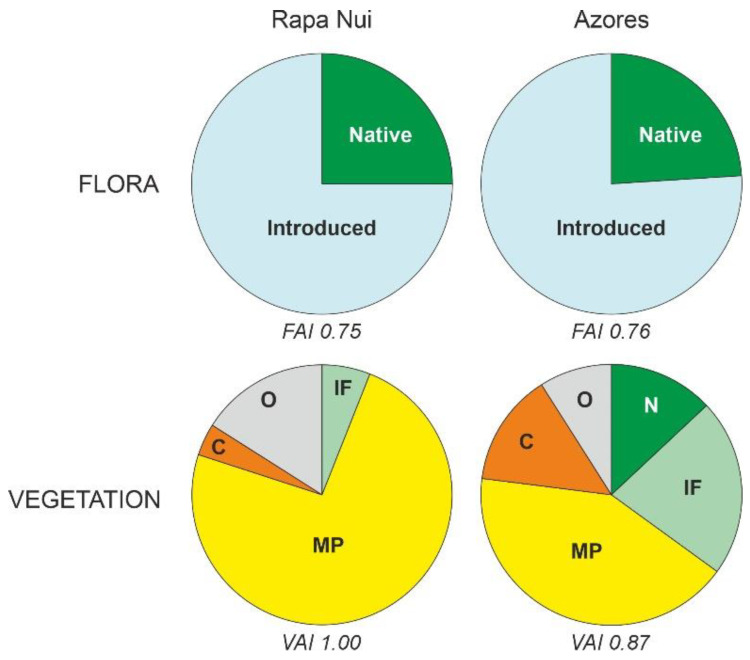
Comparison between the flora and vegetation of Rapa Nui and the Azores in terms of anthropization (data from Table 2). Vegetation types: C, crops; IF, introduced forests; MP, meadows/pastures; N, native; O, others. Anthropization indices: FAI, Floristic Anthropization Index; VAI, Vegetation Anthropization Index.

Floristically, the degree of anthropization is very similar with approximately three quarters species introduced and the others are native, in both cases (Table 2, Figure 16). The main difference lies in vegetation cover, as in Rapa Nui all extant vegetation types are introduced, whereas in the Azores native plant communities are still preserved in 13% of their total surface. Additionally, although meadows and pastures are dominant in both cases, open communities are relatively more extensive in Rapa Nui (74%) than in the Azores (42%), whereas the introduced forests are significantly reduced in the first (6%) compared with the second (22%). Croplands are also much more important in the Azores (14%) than in Rapa Nui (6%). Therefore, it could be concluded that floristic anthropization is similar in both cases (FAI 0.75–0.76), but vegetation anthropization is significantly higher in Rapa Nui (VAI 1.00) than in the Azores (VAI 0.87).

### 6.2. Early Settlement

**Figure 17:**
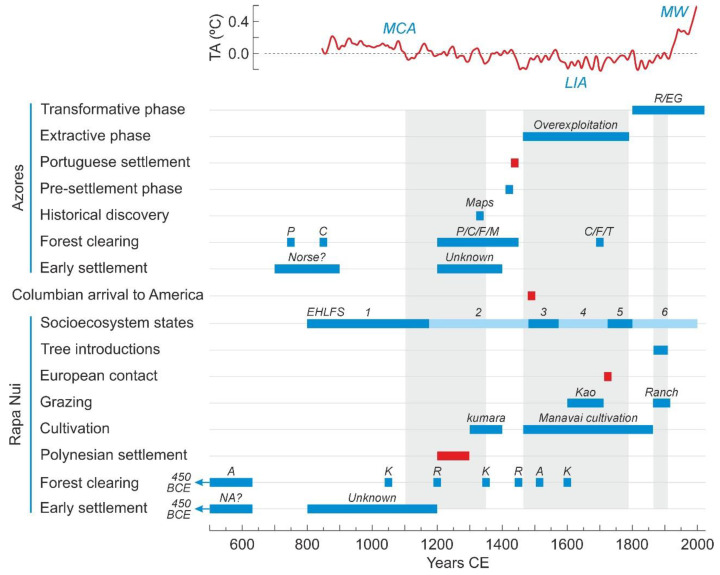
Chronological synthesis of the events and processes described in former sections for Rapa Nui and the Azores, considering the last 1.5 millennia. The gray bands highlight the phases of coincidence between Rapa Nui and the Azores trends explained in the text. Climatic phases according to Ref. [110]; TA, temperature anomaly with respect to preindustrial values. Climatic phases: L, Little Ice Age; MCA, Medieval Climate Anomaly; MW, Modern Warming. Rapa Nui localities: A, Aroi; K, Kao; R, Raraku. Azores islands: C, Corvo; F, Flores; M, São Miguel; P, Pico; T, Terceira. NA, Native Americans; R/EG, reforestation with alien trees and extensive grazing.

From the above discussion on this subject, it can be concluded that the evidence for early settlement is consistent in both Rapa Nui and the Azores. However, there is no conclusive evidence for intense and widespread anthropization in any of them before Polyensian (Rapa Nui) and Portuguese (Azores) colonization. In both cases, early settlers would have consisted of local ephemeral and/or intermittent populations practicing small-scale cultivation and animal husbandry, who did not leave archaeological remains or their traces were too faint that they were erased with time or by further permanent settlers. In Rapa Nui, early settlers would have introduced tropical American anthropochorous weeds, such as *Verbena* and *Sisyringium*, suggesting potential pre-Polynesian connections with South America, a possibility that has been supported by recent genetic analyses of other American domesticates, notably the sweet potato, and human populations. Interestingly, the most consistent and widespread paleoecological evidence for early Azorean colonizers corresponds to dates (1200–1400 CE) similar to the Polyensian settlement of Rapa Nui. This indicates that, despite the disparate geographical position in two different oceans, the different origin and cultural features of the colonizers and the different navigation technology and trajectories needed to arrive at Rapa Nui and the Azores, they were probably settled during the same time window, in the transition between the MCA and the LIA (Figure 17). The difference is that, in Rapa Nui, this settlement was permanent, whereas in the Azores, stable settlement did not occur until the Portuguese occupation (1449 CE). The origin and identity of these early settlers remains unknown. The existence of 14th-century European maps depicting some Azorean islands would suggest that these early colonizers would have been of a similar origin that further permanent settlers, but there is no empirical evidence for this hypothesis.

### 6.3. Permanent Settlement

**Figure 18:**
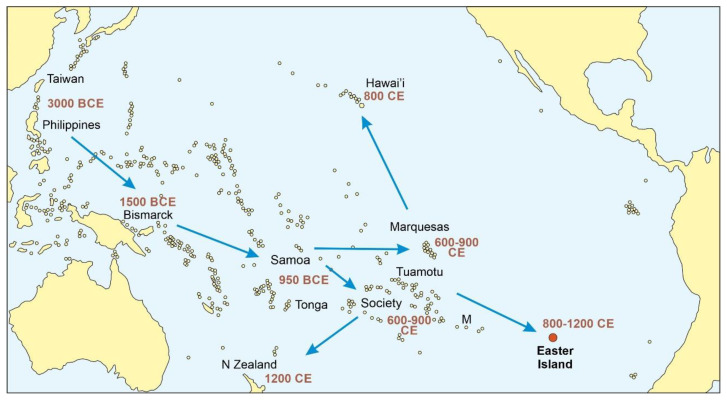
Peopling of the Pacific from East Asian archipelagos. M, Mangareva Island. Modified from Ref. [20] using raw data from Ref. [111].

The combination of archaeological and paleoecological evidence, together with paleoclimatic/paleoceanographic reconstructions agree that Polynesian settlement would have occurred approximately 1200 CE (1150–1300 CE), approximately two centuries and a half prior the Portuguese occupation of the Azores. Polynesians arrived in Easter Island after a millennial transpacific journey starting 5000 years ago (3000 BCE), when Taiwanese sailors colonized the Philippines [111]. The next step was the colonization of the Bismarck Islands, north of Papua New Guinea, by 1500 BCE. This was a fundamental stage because it implied the disconnection from the original Taiwanese culture and the development of a new culture, known as Lapita, which was the seed of all Polynesian cultures. The Lapita culture experienced a rapid expansion reaching Tonga and Samoa (western Polynesia) by 950 BCE, followed by a long pause of approximately 2000 years before the expansion toward eastern Polynesia. The archipelagos of Society, Tuamotu and Marquesas, as well as Mangareva Island, were not colonized until 600–900 CE. The last expansion wave started from these archipelagos in three different directions: Hawaii (800 CE), Easter Island (800–1200 CE), and New Zealand, the last Polynesian Island to be colonized, by 1200–1300 CE (Figure 18). Some recent studies point to a much later colonization wave (900–1200 CE) from western Polynesia (Samoa, Tonga) [97,112], and the topic is now under discussion [113].

The navigation skills of ancient Pacific peoples are well known, and developments such as the double-hulled canoe or the star-and-wind compass are outstanding examples [114]. With these and other innovations, the Polynesians and their ancestors had been able to discover the whole Pacific world from Asia to South America long before the European arrival to America. In the words of Kirch [115]: “…long before the Spanish and later the French and English, other peoples had explored the vastness of the Pacific, discovered virtually every single one of its habitable islands, and founded successful colonies on most”. The permanent settlement of the Azores was part of the maritime expansion of the Portuguese empire, which began in the early 15th century with the occupation of the western African coasts and Macaronesia and attained its maximum extension in the first quarter of the 16th century, with the funding of numerous colonies around the globe, from Brazil to Papua New Guinea, where they arrived approximately 3000 years after the Asian founders of the ancient Lapita culture. The voyage between Portugal and the Azores, separated by ~1400 km, is just under half of the journey from Mangareva to Rapa Nui (~2600 km) (Figure 18), which was the latest stage of the eastward Polynesian expansion before reaching the South American coasts [64,115]. The Polynesian and Portuguese expansions were similar in magnitude, but they diverged in that the first was exclusively Pacific and spanned millennia, whereas the second progressed mainly across the Atlantic and Indian oceans and lasted barely a century. In this context, the permanent settlement of Rapa Nui (~1200 CE) occurred approximately two centuries and a half before that of the Azores (1449 CE), which preceded the Columbian arrival to America (1492 CE) by almost half a century and the Rapa Nui European contact (1722 CE) by a couple of centuries (Figure 17).

### 6.4. Deforestation

A fundamental difference between Rapa Nui and the Azores is that the former was fully deforested by 1600 CE, whereas in the latter, the progressive decline of native forests due to their overexploitation, was compensated by a widespread reforestation (18th century onward) with alien species from diverse origins. These introduced forests have acclimated very well to the archipelago and are so dense, healthy and exuberant that they could seem pristine to an unaware visitor. Another difference is that, in Rapa Nui, forest clearing pulses did not coincide chronologically on the studied localities, whereas in the Azores, two clusters of clearing events were documented by 1200–1450 CE (before and at the beginning of Portuguese occupation) and by 1700 CE (two centuries and a half before permanent settlement), involving islands from the three geographical groups (Figure 17). The first cluster had two different phases, before and after Portuguese settlement. Before 1449 CE, at the time of early settlers, clearing pulses were usually less intense, with some exceptions, than just after permanent occupation, when native forests experienced a general, abrupt and significant decline. The 1700 CE cluster occurred when native forests were already decimated and was characterized by minor to moderate declines shortly after the general reforestation. The whole picture suggests that Azorean forests were cleared in a more homogeneous spatial fashion after Portuguese settlement. Before those dates, forest clearing pulses would have been more heterogeneous in both space and time, similar to what occurred on Rapa Nui, when different sites were deforested at different times after asynchronous local clearing events. However, early settlers never reduced native forests as intensively as Portuguese colonizers or the ancient Rapanui on their island.

### 6.5. Landscape Transformation

The progressive replacement of Rapa Nui palm forests by gardened lands coincided chronologically with the extractive phase in the Azores (Figure 17). Therefore, forest overexploitation was dominant in both cases between the 15th and 18th centuries. The main difference was the final result, which on Rapa Nui was full island deforestation, whereas in the Azores, the declining native forests were replaced by others composed of alien species from diverse origins. This Azorean reforestation began in the early 19th century and coincided, in part, with maximum vegetation deterioration in Rapa Nui, which took place during the “island ranch” phase. In both cases, this was the phase of maximum landscape anthropization, but the respective manifestations were different and have been typified as a degradation on Rapa Nui and a replacement (or landscaping) on the Azores. The introduction of allochthonous trees in Rapa Nui during the ranch period was a local and restricted practice (they occupy barely 6% of the island surface), and cannot be considered a proper replacement.

## 7. Conclusions

There is sound evidence for early ephemera/intermittent settlement in both Rapa Nui and the Azores, prior to Polynesian and Portuguese permanent settlement, respectively. In Rapa Nui, this evidence dates to a couple of millennia ago and points toward a potential Amerindian origin, as proposed by the long discredited Heyerdahl’s hypothesis. In the Azores, the evidence is much more recent (13th–14th centuries) and there is no indication of the origin of the early settlers.

According to archaeological evidence, the permanent Polynesian settlement of Rapa Nui (1200–1300 CE) would have occurred a couple of centuries prior to that of the Azores by Portuguese (1449 CE), which is well documented historically. The permanent settlement of Rapa Nui was the culmination of a millennial-scale process of occupation of virtually all Pacific islands/archipelagos from eastern Asia, whereas the occupation of the Azores occurred at the beginning of the expansion of the Portuguese empire. In this expansion, the Portuguese arrived in Papua New Guinea approximately 3000 years after the ancient Asian colonizers.

Rapa Nui was fully deforested by 1600 CE, whereas the primeval Azores forests were never totally removed and occupy a small percentage (<3%) of the total surface. The remaining total forest cover was planted during the last two centuries using allochthonous tree species from Europe, Japan and Australia, which have been successfully naturalized. The Rapa Nui deforestation was the sum of local and apparently random forest clearing pulses, whereas in the Azores, forest clearing events were of the same type at the beginning and became more widespread after permanent settlement.

Major landscape transformations occurred at similar times (15th–18th centuries) in both Rapa Nui and the Azores, and the same was true for the time of maximum landscape anthropization (19th century), which was characterized by full land degradation in Rapa Nui and widespread vegetation replacement in the Azores.

Presently, although Rapa Nui is much smaller and more isolated than the Azores, the species/area relationship is almost four times larger in the first. The percentage of endemic species is more than two times greater in the Azores. The anthropization index is almost the same if we consider the flora (three quarters of which is introduced in both cases), but is lower in the Azores if the vegetation is taken into account. Indeed, no traces of native vegetation remain on Rapa Nui, whereas in the Azores, it occupies approximately 13% of the total surface.

A summary of the similarities and differences is provided in Table 3. In general, most similarities correspond to the time after permanent human settlement of both Rapa Nui and the Azores, whereas the differences are more widespread over the pre and post-settlement phases (see also Figure 17).

## 8. Prospects for Future Research

Much work is still to be done for a thorough assessment on human settlement and landscape transformation in Rapa Nui and the Azores. Early settlement is a key point and, although it is consistently supported by the available evidence, its spatiotemporal patterns remain largely unknown and need further paleoecological, archaeological and anthropological research for a sound assessment. In Rapa Nui, the use of molecular biomarkers for human presence and activities is still lacking and could be instrumental in clarifying the origin and cultural features of potential early settlers. In the Azores, more records encompassing the last millennia across the whole archipelago are still needed for an archipelago-wide appraisal. Another important aspect that remains hypothetical is the influence of environmental (mainly climatic) changes on the studied island socioecosystems. The available paleoclimatic and paleoecological records are supportive of meaningful environment-human-landscape feedbacks and synergies, but the finding of new records and the use of novel proxies are needed for an unequivocal assessment and the establishment of causal relationships. Paleoclimatic trends of the last millennia remain largely unknown or are rather contradictory in both Rapa Nui and the Azores, and further research is needed to clarify this point. The development of new lake/swamp coring campaigns is encouraged to establish a coherent paleoenvironmental framework to be compared with the available archaeological, anthropological and historical information in the way toward a holistic view using human ecodynamic approaches such as the EHLFS or similar approaches [104]. This initiative should be developed by multidisciplinary teams including scholars from a diversity of scientific fields, rather than by one or a few specialized researchers or research teams biased toward certain specialties [20]. The development of a global database on the spatiotemporal patterns of settlement and anthropization of oceanic islands would be useful for worldwide comparisons based on the criteria used in this paper and others similar [49].

## Figures and Tables

**Table 1:** Terminology used in this paper. Adapted and expanded from Ref. [20].

Term	Meaning	Types	Meaning	Evidence (Examples)
Discovery	Finding the island for the first time	Sighting	See the island in the distance	Historical (maps, logbooks)
		Landing	Physically arrive on the island	
Settlement	Physical occupancy of the island	Ephemeral	Temporary occupancy	Historical (written documents); archaeological (housing, monuments, art); paleoecological and archaeobotanical (biomarkers, cultivated plants, fire); anthropological (DNA)
		Intermittent	Recurrent occupancy and abandonment	
		Permanent	Long-lasting occupancy	
		Local	Restricted to one or a few sites	
		Full	Extensive to the whole island	
Contact	Arrival of a new culture to an already settled island	Visit	Stopover with or without cultural incidence	Historical (written documents); archaeological (housing, monuments, art); anthropological (fossil skeletons, DNA, ethnography)
		Admixture	Cultural/biological merging	
		Invasion	Violent occupation or slavery practices	

**Table 2:** Main traits of the Rapa Nui and Azores flora and vegetation in relation to anthropization. The category “others” in vegetation types includes urban areas, unvegetated terrains and badlands. FAI (Floristic Anthropization Index) is the proportion of anthropogenic (introduced) vs. native species, ranging from 0 (pristine) to 1 (fully anhthropized). VAI (Vegetation Anthropization Index) uses the same calculation applied to the percentage of natural vs. anthropogenic vegetation cover. Raw data from the above sections.

		Rapa Nui	Azores
Extent	Total surface (km^2^)	164	2351
Isolation	Nearest continent (km)	3700	1400
	Nearest island (km)	2100	900
Vascular flora	Total species (n)	201	811
	Species/area	1.22	0.34
	Native (%)	25	24
	Introduced (%)	75	76
	Endemics (%)	12	30
Vegetation cover	Native types (%)	0	13
	Introduced forests (%)	6	22
	Meadows/pastures (%)	74	42
	Crops (%)	4	14
	Others (%)	16	9
Anthropization	FAI	0.75	0.76
	VAI	1	0.87

**Table 3:** Similarities and differences between Rapa Nui and the Azores in terms of human settlement and landscape anthropization, as discussed in the text. RN, Rapa Nui; AZ, Azores.

Process	Similarities	Differences
Early settlement	Consistent evidence (forest clearing, fire, cultivated plants/weeds)	Timing and origin: RN—Amerindian component (450 BCE) AZ—Unknown origin (13th–14th centuries)
Permanent settlement	Timing (1200–1450 CE)	Origin and expansion routes: RN—Ancient Polynesians (late eastward expansion) AZ—Modern Portuguese (early westward imperial expansion)
Forest clearing	Overexploitation and time of initiation (15th century)	Intensity: RN—Full deforestation (1600 CE) by random local pulses AZ—Partial deforestation by generalized events
Landscape transformation	Timing of major transformations (15th–18th centuries) and maximum anthropization (19th century)	Type of transformation: RN—expansion of gardening cultivation and extensive grazing AZ—extractive practices and further reforestation (18th century)
Present conditions	Floristic anthropization (75%)	Species/area relationships: RN—1.22; AZ—0.34 Endemic species (%): RN—12%; AZ—30% Vegetation anthropization: RN—1.00; AZ—0.87
